# Structural and Phylogenetic Analysis of Laccases from *Trichoderma*: A Bioinformatic Approach

**DOI:** 10.1371/journal.pone.0055295

**Published:** 2013-01-31

**Authors:** Saila Viridiana Cázares-García, Ma. Soledad Vázquez-Garcidueñas, Gerardo Vázquez-Marrufo

**Affiliations:** 1 Centro Multidisciplinario de Estudios en Biotecnología, Facultad de Medicina Veterinaria y Zootecnia, Universidad Michoacana de San Nicolás de Hidalgo, Morelia, Michoacán, Mexico; 2 División de Estudios de Posgrado, Facultad de Ciencias Médicas y Biológicas “Dr. Ignacio Chávez”, Universidad Michoacana de San Nicolás de Hidalgo, Morelia, Michoacán, Mexico; Cinvestav, Mexico

## Abstract

The genus *Trichoderma* includes species of great biotechnological value, both for their mycoparasitic activities and for their ability to produce extracellular hydrolytic enzymes. Although activity of extracellular laccase has previously been reported in *Trichoderma* spp., the possible number of isoenzymes is still unknown, as are the structural and functional characteristics of both the genes and the putative proteins. In this study, the system of laccases *sensu stricto* in the *Trichoderma* species, the genomes of which are publicly available, were analyzed using bioinformatic tools. The intron/exon structure of the genes and the identification of specific motifs in the sequence of amino acids of the proteins generated *in silico* allow for clear differentiation between extracellular and intracellular enzymes. Phylogenetic analysis suggests that the common ancestor of the genus possessed a functional gene for each one of these enzymes, which is a characteristic preserved in *T. atroviride* and *T. virens*. This analysis also reveals that *T. harzianum* and *T. reesei* only retained the intracellular activity, whereas *T. asperellum* added an extracellular isoenzyme acquired through horizontal gene transfer during the mycoparasitic process. The evolutionary analysis shows that in general, extracellular laccases are subjected to purifying selection, and intracellular laccases show neutral evolution. The data provided by the present study will enable the generation of experimental approximations to better understand the physiological role of laccases in the genus *Trichoderma* and to increase their biotechnological potential.

## Introduction

Laccases (benzenediol:oxygen oxidoreductase, EC 1.10.3.2) are metalloenzymes that belong to the multicopper oxidase (MCO) family. These enzymes catalyze the oxidation of various aromatic substrates with the concomitant reduction of molecular oxygen to water. This redox process is mediated by two centers that contain four atoms of copper in their +2 oxidation state. These copper atoms are classified as T1 (blue copper), T2 or T3 according to their spectroscopic characteristics [1]. Laccases are generally monomeric glycoproteins with molecular weights that range from 60 to 70 kDa, and up to 30% of their molecular weight is made up of carbohydrates [2]. These enzymes are widely distributed in nature, and the physiological functions that they perform depend both on their origin and on their biochemical and structural properties. In fungi, laccase activities have been related to the degradation of lignocellulose material, the production of pigments, sporulation, processes of morphogenesis, phenomena of pathogenesis toward plants and animals [3], the oxidation of antibiotics produced by microorganisms that are antagonists of plant pathogens and antimicrobial components of plants, such as flavonoids or phytoalexins [4]. This great functional versatility is partly due to the fact that laccases possess low substrate specificity and exhibit a broad range of redox potentials [2]. Because of this flexibility, these enzymes are able to act on ortho- and para-diphenols, methoxy-substituted phenols, aromatic diamines and benzenothiols. Furthermore, these enzymes can oxidate organic and inorganic metallic compounds. In addition, the gamut of substrates for laccases can extend to non-phenolic compounds through the inclusion of redox mediators, with which they are able to oxidize large polymers, such as lignin, cellulose or starch [5]. This peculiarity has been exploited by various biotechnological processes, including biopulping, bioremediation, the breakdown of colorants, the enzymatic conversion of chemical intermediates and the synthesis of pharmaceutical products, among others [6].

The majority of laccases used in biotechnology are derived from fungal species. The presence of laccases has been documented in various groups of fungi, including yeasts [7], filamentous ascomycetes [8] and white [9] and brown rot fungi [10], as well as mycorrhizal species [11]. In general, until now it has been found that fungi produce more than one laccase enzyme, the expression of which are closely related to environmental conditions or the stage of the life cycle and lifestyle of the fungus [12], [13]. For these reason, these enzymes are synthesized in variable quantities, which makes the identification of the complete laccase system in a single species difficult. The characterization of families of laccase genes has progressed due to the availability of genomic sequences. Tblastn analysis has allowed for the definition of the complete number of laccase genes and their corresponding proteins in both basidiomycete [11], [14], [15] and ascomycete fungi [8], [12].

Species of the genus *Trichoderma* are characterized by rapid growth, the ability to assimilate a large variety of lignocellulose substrates and resistance to toxic chemical products [16]. Several species in the genus, particularly *T. reesei/Hypocrea jecorina*, are good producers of extracellular enzymes that degrade plant cell walls, such as cellulases and hemicellulases, for which reason they have been used in the production of recombinant proteins at industrial levels. Other species, such as *T. harzianum/H. lixii, T. virens/H. virens* and *T. atroviride/H. atroviridis,* are used as biological control agents against fungal pathogens of plants and nematodes [17].

Extracellular laccase activity has been detected in various strains of *Trichoderma* spp., including isolates not identified at the species level [18] as well as distinct strains of *T. viride, T. reesei, T. atroviride* and *T. longibrachiatum* [19], [20]. Laccase activity associated with the conidia of *T. atroviride, T. viride* and *T. harzianum* has also been documented. In these strains, it is hypothesized that the enzyme is found in the membrane or in the periplasmic space [21], [22]. Recently, the purification and characterization of extracellular laccases in wild strains of *T. harzianum* [23], *T. atroviride* [24] and *T. reesei* [25] have been documented. Catalano *et al.* [26] have evaluated the role of an extracellular laccase from *T. virens* in the mycoparasitism of that species against the sclerotia of the phytopathogens *Botrytis cinerea* and *Sclerotinia sclerotiorum.*

Despite the fact that currently there are access to the complete sequence of the genomes of *T*. *atroviride, T*. *virens* [27], *T. reesei* [28], *T. harzianum* (http://genome.jgi-psf.org/Triha1/Triha1.home.html) and *T. asperellum* (http://genome.jgi-psf.org/Trias1/Trias1.home.html), at this date, an *in silico* analysis has not been performed that would characterize the number of genes coding for laccase activity in these species and the structural characteristics of the coded proteins.

It has been documented that laccases *sensu stricto* from asomycetes has a number of signatur characteristics not present in laccases from basidiomycetes. These signatures that are additional to the L1–L4 domains [29] and allow differentiate such proteins from other Multi-copper oxidases (MCOs), includes an SDS-gate [30], a C-terminal DSGL/I/V domain [31], and the presence of a F/L residue in axial coordination of the T1 copper [32]. Although two of the studies cited above included a search to detect the presence of laccases in some of the genomes available for the species of the *Trichoderma* genus [25], [26], a comparative analysis of the identified genes to elucidate the number of laccases *sensu stricto*, the relationships between them, their possible cellular localization and their putative functions has not been performed. This analysis constitutes the principal objective of the present study.

## Materials and Methods

From the NCBI GenBank database, we obtained the sequences of various multicopper oxidases, including those of *Saccharomyces cerevisiae* (Fet3p, 763529), *Melanocarpus albomyces* (Laccase, 40788173), *Cucurbita maxima* (Ascorbate oxidase, 885589) and *Myrothecium verrucaria* (Bilirubin oxidase, 456712), which were used as queries to search for laccase genes in species of *Trichoderma*. Various members of the family of MCOs were used to assure the identification of all possible laccases in the genomes analyzed based on the identity of copper binding sites. In addition, only the genes and sequences of amino acids from crystallized proteins were used, for which there is no doubt regarding their identity. A Blastp/Blastn analysis was performed on the database of the public genomes of *T. asperellum* (http://genome.jgi-psf.org/Trias1/Trias1.home.html), *T. atroviride* (http://genome.jgi-psf.org/Triat2/Triat2.home.html), *T. harzianum* (http://genome.jgi-psf.org/Triha1/Triha1.home.html), *T. virens* (http://genome.jgi-psf.org/TriviGv29_8_2/TriviGv29_8_2.home.html) and *T. reesei* (http://genome.jgi-psf.org/Trire2/Trire2.home.html). Sequences were selected for the presence of the four preserved motifs of copper-binding characteristic of all MCOs.

To analyze the structural characteristics of *Trichoderma* spp. laccases, the online programs of the Center for Biological Sequence Analysis (CBS) (http://www.cbs.dtu.dk/services/) were used. The programs SignalP Version 4.0 and PrediSi were used to determine the presence of the peptide signal for secretion and putative cleavage sites, whereas NetNGlyc 1.0 was used to determine the sites of N-glycosylation (Asn-XXX-Ser/Thr). For those proteins that were classified as intracellular, we used the packages TargetP Version 1.1, iPSORT (http://ipsort.hgc.jp/) and MitoProt (http://ihg.gsf.de/ihg/mitoprot.html) to establish their putative subcellular localization. The position and composition of the cupredoxin domains were analyzed in SWISS-MODEL (http://swissmodel.expasy.org/).

For the phylogenetic analysis of the putative sequences of laccase, multiple alignment was performed with CLUSTALX Version 2.0.11 (http://www.clustal.org/clustal2/) using the predetermined parameters. The sequences used for phylogenetic analysis with their respective accession numbers for GenBank and JGI genome portal are presented below (the key used in this article for each species appears in parentheses). **Ascomycetes**: *Ajellomyces dermatitidis* (Ade) 327349048; *Arthroderma gypseum (*Agy) 315039999; *Aspergillus niger* (Ani) fge1_e_gw1_12.409, e_gw1_4.1637, gw1_10.607; *Botriotynia fuckeliana* (Bfu) 15022487, 15022489, 347830053; *Botrytis aclada* (Bac) 378942783; *Chaetomium globosum* (Cgl) CHGG03552.1, CHGG02290.1, CHGG10025.1, 06172.1; *Colletotrichum lagenarium* (Cla) 12862766; *Cryphonectria parasitica* (Cpa) 167469, 69047730*; Fusarium oxysporum* (Fox) 152013644; *Gaeumannomyces graminis* var. *graminis* (Ggg) 19309738, 19309740; *Gaeumannomyces graminis* var. tritici (Ggt) 19171197, 19171195, 19171193; *Hortaea acidophila* (Hac) 63146072, 67773582; *Magnaporthe oryzae* (Mor) 389627974; *Melanocarpus albomyces* (Mal) 40788173; *Monilinia fructigena* (Mfr)120431232; *Myceliophthora thermophila* (Mth) 367028915; *Myrioconium* sp. (Msp) 160332827; *Neurospora crassa* (Ncr) NCU9279.5, NCU05604.5, NCU05113, NCU04528, NCU02201.5, NCU07920.5, NCU09023.5, NCU00526.5; *Phoma* sp. (Psp) 166812033; *Podospora anserina* (Pan) Pa_7_4200, Pa_5_4660, Pa_5_1200, Pa_5_9860, Pa_6_7880, Pa_7_3560, Pa_1_15470; Pa_1729781, Pa_5_4140; *Pyrenophora tritici-repentis* (Ptr) 189188518, 189192570; *Sclerotinia minor* (Smi) 120431228; *Sclerotinia sclerotiorum* (Ssc) 156056931; *Sordaria macrospora* (Sma) SMAC09326, SMAC06098, SMAC03641, SMAC03042, SMAC09228, SMAC09572, SMAC01222; *Sporotrichum thermophile* (Sth) 99853; *Thielavia arenaria* (Tar) 333361328; *Thielavia terrentris* (Tte) 2011187; *Trichoderma asperellum* (Tas) 154312, 71665, 68620; *Trichoderma atroviride* (Ta) 54145, 40409; *Trichoderma harzianum* (Th) 539081; *Trichoderma reesei* (Tr) 122948; *Trichoderma virens* (Tv) 48916, 194054; *Yarrowia lipolytica* (Yli) 340748006. **Basidiomycetes**: *Agaricus bisporus* (Abi) 2833227, 2833228; *Coprinopcis cinerea* (Cci) 42721544, 4838342, 4838344, 4838346; *Coriolopsis gallica* (Cga) 12484399*; Coriolus hirsutus* (Chi) 167465; *Gelatoporia subvermispora* (Gsu) 31088842; *Lentinula edodes* (Led) 18146854, 18146856, 6466812; *Marasmius quercophilus* (Mqu) 6318611; *Pleurotus ostreatus* (Pos) 15594026, 2833235, 2833237, 3006039; *Polyporus ciliatus* (Pci) 9957143, 9957145, 9957147; *Pycnoporus cinnabarinus* (Pcn) 10179427, 3128389, 5732664; *Trametes pubescens* (Tpu) 20270770; *Trametes* sp. (Tsp) 21616730, 56785436; *Trametes trogii* (Ttr) 119416759; *Trametes versicolor* (Tve) 15778442, 2388517, 2598857; *Trametes villosa* (Tvi) 2842752, 2842753; *Volvariella volvacea* (Vvo) 42416980. **Plant**: *Arabidopsis thaliana* (Ath) 332191557; *Rhus vernicifera* (Rve) 19912797. Redundant sequences, that is, those from the same species with an identity greater than 95%, were discarded.

The alignments obtained were manually adjusted. Based on the generated alignments, phylogenetic trees were constructed with MEGA Version 5.05 (http://megasoftware.net/) through the Neighbor Joining method using three different models of evolutionary distance (p-distances, Dayhoff and Jones-Taylor-Tornton). Statistical significance was evaluated with a bootstrapping of 1000 repetitions. The phylogenetic trees were confirmed using the maximum likelihood method (data not shown), and the alignments were differentially edited to corroborate the topology of the obtained trees.

A phylogram of the analyzed species of *Trichoderma* was constructed using the *rpb2* gene (coding for RNA polymerase B II) through Bayesian analysis in accordance with [27]. The rates of synonymous and non-synonymous substitutions of *Trichoderma* laccases in the nucleotide sequences aligned by codons were calculated with the SNAP package (www.hiv.lanl.gov/content/sequence/SNAP/SNAP.html).

## Results and Discussion

### Number and Structure of Laccase Genes in *Trichoderma*

To determine the number of laccase genes in the five analyzed species of *Trichoderma,* we conducted a Blastp/Blastn analysis of the database of the genome of each species. The search produced a total of 47 sequences that presented the four copper binding motifs characteristic of MCOs. In *T. reesei,* a total of 7 genes were identified; in *T. harzianum,* 9 genes; *T. atroviride* and *T. virens* presented 10 genes each, and in *T. asperellum,* 11 genes were found. To determine which of the identified genes coded for laccases *sensu stricto,* the structural characteristics previously reported in the literature that distinguish laccases from other copper blue oxidases were sought based on a comparative analysis of laccase sequences and crystallographic evidence (Table 1). Based on this analysis, a single gene coding for laccases was found in *T. ressei* and *T. harzianum*, two genes were found in *T. atroviride* and *T. virens* and three genes in *T. asperellum* (Table 2); the rest of the identified genes belong to other members of the MCO family and were not considered in further analyses.

**Table 1:** Observed signature sequences in laccases.

Signature		Reference
**Axial coordination**	Leu or Phe	[32]
**L1**	H-W-H-G-X9-D-G-X5-QCPI	[29]
**L2**	G-T-X-W-Y-H-S-H-X3-Q-Y-C-X-D-G-L-X-G-X-(FLIM)	
**L3**	H-P-X-H-L-H-G-H	
**L4**	G-(PA)-W-X-(LFV)-HCHI-DAE-X-H-X3-G-(LMF)-X3-(LFM)	
**SDS gate**	Ser143, Ser511 and Asp561 in TaLcc1	[42]
**C-termini**	Asp-Ser-Gly-(Leu/Ile/Val)	[31]

In the fungal laccase signature sequences L1–L4, an X represents an undefined residue, whereas the multiple residues within brackets represent a partially conserved residue.

**Table 2:** Accession numbers and structural characteristics of laccases *sensu stricto* from *Trichoderma* spp.

Species	ID^a^	Length precursor	Signal P^b^	N-glycosylationAsn-X-Ser/Thr^b^	MW^c^ (kDa)	p*I* ^d^
***T. asperellum***	154312	566	VLA-FP	N91, N120, N132, N232, N245, N273, N309, N409, N544	62.05	4.35
	68620	590	nd	nd	64.90	6.25
	71665	600	AVA-LS	N36, M85, N129, N320, N351, N351, N421, N425, N455, N472, N508	66.34	5.43
***T. atroviride***						
	54145	566	VYA-FP	N91, N120, N132 N232, N245, N273, N290, N309, N402, N544	61.83	4.44
	40409	590	nd	nd	65.21	6.51
***T. harzianum***	539081	589	nd	nd	65.16	4.97
***T. reesei***						
	122948	568	nd	nd	63.37	6.06
***T. virens***						
	48916	566	VCA-IP	N91, N120, N132, N232, N245, N273, N309, N402, N412, N426, N544	62.14	4.32
	194054	588	nd	nd	65.23	6.49

a ID, identification number in the *Trichoderma* species genome; ^b^Denotes the location of signal peptide cleavage sites in amino acid sequences from *Trichoderma* laccases; nd, non detected. ^c^MW, estimated molecular weight.^d^p*I*, predicted isoelectric point.

The number of laccase genes in ascomycete fungi varies considerably. Among the species that are characterized by having a larger number of laccase genes are *P. anserina* and *S. macrospora* with 9 genes [12], *N. crassa* with 8 [12], *A. niger* with 6 [8], and *C. globosum* with 4 [13]. Among the species characterized by having a low number of laccase genes are *G. gramminis* with 3 genes in *var. tritici* [33] and 2 in *var*. *gramminis* [33]. Yeasts are a particular case within the ascomycetes, as it has been reported that they do not have laccase genes [13], although the presence of 2 genes [7] has been documented in *H. acidophila*. Thus, *Trichoderma* belongs within the group of fungi with a low number of laccase genes. Nevertheless, the results of the structural analysis performed in the present study indicate that the laccase genes previously reported in ascomycetes should be reviewed in the future, as it is possible that several of them do not code for *sensu stricto* laccases (see below).

To determine the relative position of the laccase genes in the genomes of all *Trichoderma* species, approximately 15 kb upstream and downstream regions were analyzed in each case. This analysis shows that the different laccase encoding genes within the same genome of the analyzed species are not arranged in clusters, but are far from each other. It was also found that the genomic context of the corresponding orthologous genes is similar between all analyzed species (data not shown). In general, the genes encoding for intracellular laccases show a higher synteny than the extracellular ones. To date, there are no data in the literature that allow us to compare the relative position of laccase genes for ascomycetes. However, the arrangement of the laccase genes in *Trichoderma* is consistent with what was found in the basidiomycete *L. bicolor*, where most laccases encoding genes are randomly distributed [11]. Nevertheless, the existence of laccase gene clusters has been observed in *C. cinerea* [14] and *P. ostreatus* [15]. These differences in genomic architecture indicate that this type of genes does not have a conserved location within fungal genomes but that their disposition reflects the evolutionary history of each species.

**Figure 1:**
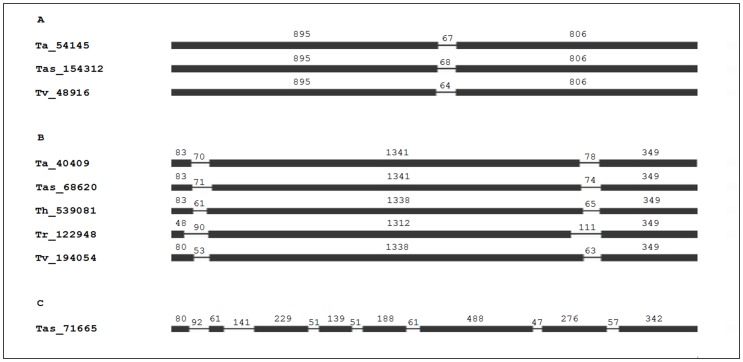
Intron positions within the laccase genes of *Trichoderma* spp. define three gene subfamilies. The thin lines indicate intron positions and the black lines indicate the exons. The first subfamily (**A**) contains only one intron, the second subfamily (**B**) contains two introns and the final (**C**) subfamily contains seven introns.

The nucleotide sequence length of *Trichoderma* spp. laccase encoding genes varies between 1765 (Tas_154312) and 2303 bp (Tas_71665). The GC percentage of these genes ranges from 46% (Tas_71665) to 58% (Tr_122948). The number and position of introns in laccase encoding genes in fungi have been employed to classify them into subfamilies [11], [14]. The structure of the genes found in the five species of *Trichoderma* separates them into three subfamilies. In each subfamily, the number of introns is preserved, and their positions are similar (Fig. 1). The first subfamily exhibits an intron between 64 and 68 bp in size and is made up of a gene of each one of the species *T. atroviride, T. asperellum and T. virens* (Fig. 1A). The second subfamily is characterized by having two introns of between 53 and 111 bp, with one of these genes being found in the five species of *Trichoderma* (Fig. 1B). In general, the subdivision of *Trichoderma* laccase encoding genes in these two subfamilies is in agreement with previous reports of a low number of introns in this type of genes in the ascomycetes. In two varieties of *G. gramminis,* the gene sequences of *LAC1* and *LAC2* present 2 introns [33], just as in the single gene for extracellular laccase reported for the aquatic species *Myrioconium* sp. [34]. The presence of 3 introns has been reported in the *lac2* gene [35] in the case of *P. anserina* and in the *Bclcc1* and *Bclcc2* genes of *B. cinerea* [4]. In the yeast *H. acidophila,* 3 introns have been documented in the gene that encodes for the extracellular enzyme and 2 for the intracellular enzyme gene [7]. A unique case is that of the Tas_ 71665 gene of *T. asperellum*, with a structure of seven introns and for which no orthologous genes were found in the other analyzed *Trichoderma* species (Fig. 1C). The only ascomycetes laccase gene that surpasses this number is the *lac-1* gene of *C. parasitica*, which possesses 12 introns [36]. These two last genes represent intermediaries in the structure of introns/exons between the majority of ascomycetes and the basidiomycetes *C. cinerea* and *L. bicolor*, in which two subfamilies of genes have been found whose number of introns varies between 13 and 15 [11], [14]. The introns found in the *Trichoderma* laccase genes preserve the splicing sites that comply with the GT/AG rule [37]. Previous data show the usefulness of the number and location of introns for generating subfamilies of ascomycetes laccase genes. This information could be useful for the identification of genes with distinct functions within a single species or for recognizing the same gene in different species. This analysis is beyond the scope of the present study and should be conducted in greater detail in the future. Interestingly, as a demonstration of the usefulness of gene family classification based on the presence of introns, the subfamilies of laccase genes formed in *Trichoderma* agree with classes established according to laccase protein structural characteristics (see below).

### Structural Characteristics of *Trichoderma* Laccases

In general, the identification of laccases according to their amino acid sequence involves the recognition of the four segments L1–L4 [29]. However, such regions are common to all MCO family members including ascorbate oxidases, ferroxidases and bilirubin oxidases, meaning that their presence is not sufficient to confirm that a protein sequence corresponds to a laccase *sensu stricto*. Because of this finding, in the present study we also considered the SDS-gate and the C-terminus end, which are distinctive characteristics of ascomycetes laccases (Table 1). The set of structural characteristics found in the amino acid sequence of *Trichoderma* laccases is detailed below.

**Figure 2:**
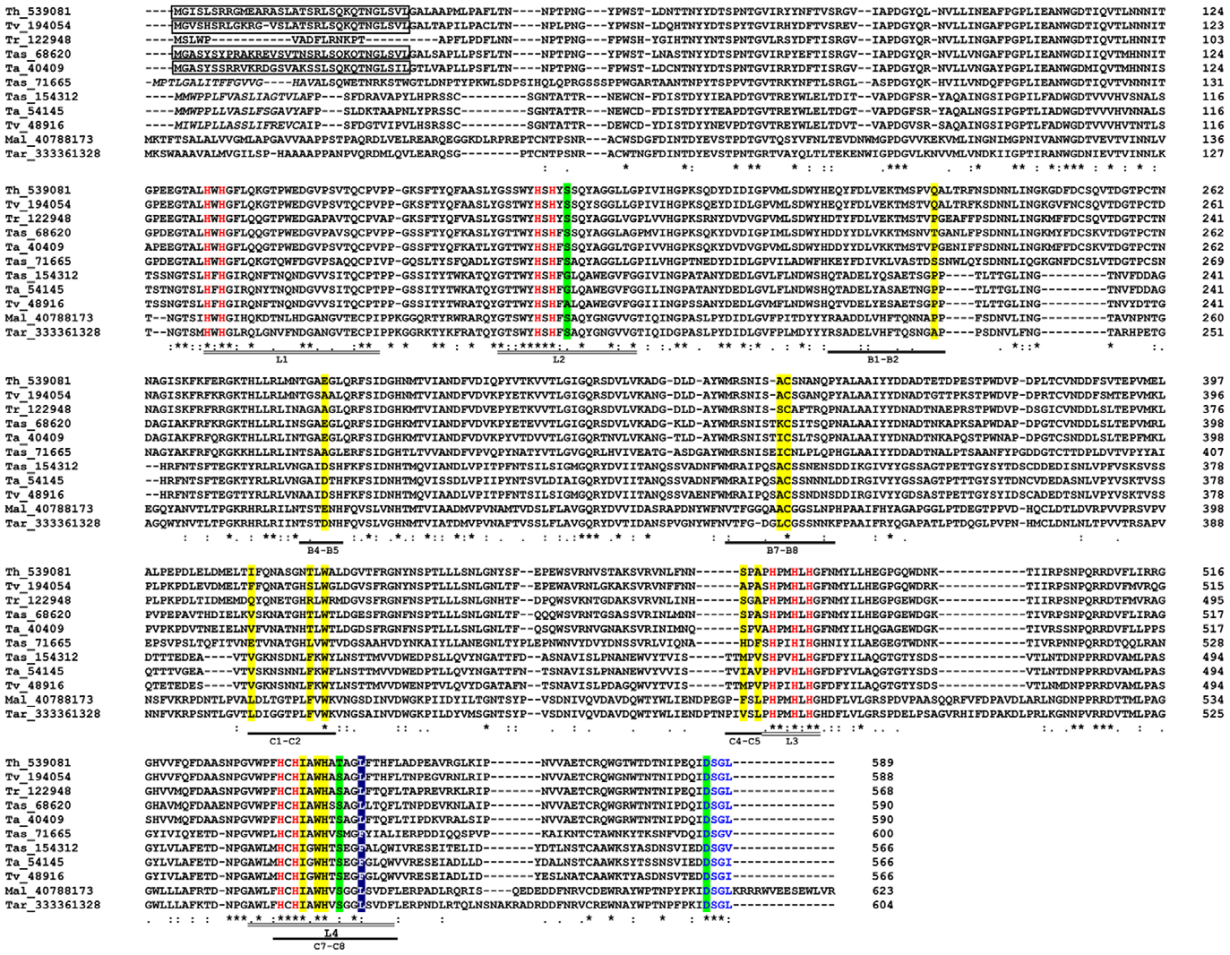
Alignment of laccase sequences from *Trichoderma* spp. The alignment was constructed with the Clustal X multiple-sequence alignment program. The accession number of each sequence in the JGI GeneBank is indicated on the left of the alignment. An asterisk indicates that the residues at a position are identical in all sequences in the alignment; a colon indicates that conserved substitutions have been observed and a period indicates semiconserved substitutions. Putative signal sequences are indicated by italics and the mitochondrial targeting peptides are enclosed in boxes. The conserved residues involved in copper binding are in red, and the complete L1–L4 regions are indicated by a double line under the alignment. The sequences of potential substrate loops were identified based on loops I-IV of the rMaL [44] and TaLcc1 [42], laccases of *M. albomyces* and *T. arenaria*, respectively, and are underlined with a bold line. Amino acids shaded in yellow indicate residues in contact with the substrate. The residues forming the SDS gate are shaded in green in color, and the amino acid shaded in blue classified the laccases as class 1 (Met), class 2 (Leu) or class 3 (Phe). The conserved C-termini are in dark blue.

The analysis of the amino acid sequences of the 9 putative laccases that were found shows that their lengths are similar to the typical laccases of fungi (566–600 aa), and the calculated molecular mass for the protein sequences is between 61.83 and 66.84 kDa with acidic isoelectric points (Table 2). These results are in agreement with what has previously been reported for fungal laccases, which regularly have molecular weights between 60 and 70 kDa and which have isoelectric points (p*I*) that vary between pH 4.0–6.0 [3]. In particular, within the genus *Trichoderma*, purified laccases from the wild strains WL1 of *T. harzianum* and CTM 10476 of *T. atroviride* presented, in their glycosylated form, molecular weights of 79 and 80 kDa, respectively [23], [24]. Analysis in SWISS-MODEL of the proteins found in the genomes reviewed in this study showed that each one of the sequences is formed by three cupredoxin domains that are ordered in sequential form and are common to all the MCO family members [30]. As expected, the residues of amino acids that bind to copper T1 were located in domain I, whereas the residues that coordinate coppers T2/T3 were distributed between domains I and III (Fig. 2).

Laccases are distinguished by the presence of four consensus sequences, L1–L4, which possess a length of between 8 and 24 amino acid residues and are distributed along the polypeptide chain. Within these regions, one finds amino acid residues that serve as ligands for the copper atoms, as well as other preserved or partially preserved residues, which are critical for maintaining the conformational folding of the enzyme. Such characteristic sequences are found based on the multiple alignment of more than 100 laccases of plants and fungi [29] and represent the distinctive mark for the identification of putative new laccases. Regions L1–L4 in the *Trichoderma* sequences have a high degree of similarity with the consensus designated by [29] (Fig. 2). However, some amino acid residues differ from the consensus. This finding is especially evident in L2, where Thr is replaced by a Ser in the second position. This change of amino acid is found in the Lac2 of *G.graminis* var. *tritici* and *G. graminis* var. *graminis* [33], as well as in the Lac1 of *B. fuckeliana* (anamorph = *B. cinerea*) [4], both of which are ascomycete fungi. In this same segment, in *Trichoderma,* changes in the consensus QYCDGL are observed: Tyr is replaced by Ala, Cys by Ser/Ala/Trp, Asp by Gly/Glu and Leu by Val (Fig. 2). Thus, the results obtained here show that although laccases of basidiomycetes fully comply with the consensus designated by L2 [15], this region varies considerably in ascomycete laccases. In segments L1 and L4 of the *Trichoderma,* gene changes that involve residues of amino acids with propensities toward similar conformations or similar hydropathic indices are observed. For example, in L1, Trp is replaced by Phe (Fig. 2). This same change is observed in *C. parasitica* Lac3 [38]. In segment L4, the amino acid located 10 residues downstream from the preserved Cys corresponds to the axial position of copper T1. This residue is usually a Met in the MCOs; however, in the laccases, the Met residue is replaced by a Leu or Phe [39], as in the *Trichoderma* spp. putative laccases (Fig. 2).

Axial coordination is one of the factors affecting the redox potential (*E_0_*) of laccases [39]. It has been previously suggested that laccases with high *E_0_* (700–800 mV) possess a Leu or Phe in axial coordination, whereas laccases with a Met residue have low *E_0_* (500 mV). Based on the substitution of this residue, laccases are classified into three classes: Lac 1 (M, Met), Lac 2 (L, Leu) and Lac 3 (F, Phe) [32]. Taking this characteristic into account, the putative laccases Ta_40409, Tas_68620, Th_539081, Tr_122948 and Tv_194054, are classified as Lac 2, whereas Tas_71665, Tas_154312, Ta_54145 and Tv_48916 are classified as Lac 3. These results are consistent with the hypothesis that Lac 1 laccases are primarily present in plants [32]. This classification has been performed in one isoenzyme of each of the ascomycetes: *C. parasitica, N. crasa*, and *P. anserina*, belonging to class 2 [32]. Although this classification is still used to elucidate the functional relatedness between laccases, it is important to take into account that crystallographic data obtained from native high potential laccase of *T. versicolor* (TvL) have helped to describe other structural characteristics that might contribute to high *E_0_* in these enzymes [40]. Such structural features include a reduction of electron density in the metal and the ligating amino acid. In high redox potential laccases, the distance between Cu^+2^ and one of the histidines from the T1 binding pocket is longer compared with that in enzymes of the middle potential group; the hydrogen bond between Glu-460 and Ser-113 in TvL seems to be responsible for this [40]. Interestingly, this particular serine is conserved in *Trichoderma* laccases, but the Glu residue is not present. Furthermore, it is well known that several factors can affect the *E_0_* value of metalloproteins, including electrostatic intramolecular interactions, and solvation effects [41]. It is important to conduct further studies that allow for determining those factors involved in the modulation of redox potential in *Trichoderma* laccases.

Laccases have been the subject of intense investigation directed at understanding both their catalytic mechanism and the molecular determinants that modulate their broad range of *E_0_* [40]. Although the laccase reaction scheme is not entirely understood, it is known that both binding and the oxidation of the substrate occurs in the T1 site and the electrons are transferred to the T2/T3 center, where the reduction of molecular oxygen takes place [40]. The reduction of a dioxygen molecule to two water molecules requires four electrons and four protons. The electron transfer pathway to the trinuclear center corresponds to the preserved motif Hys-Cys-Hys located at L4 [39], which is present in *Trichoderma* laccases (Fig. 2). Conversely, proton transfer is assisted by the so-called SDS-gate [30]. This gate is formed by two residues of Ser and one of Asp and is conserved in ascomycete laccases but has not been detected in basidiomycete laccases. In *T. arenaria* laccase TaLcc1, this gate is formed by Ser143, Ser511 and Asp561 [42]. Multiple alignment with TaLcc1 identified the SDS-gate amino acids in *Trichoderma* spp. laccases (Fig. 2). However, in Tas_154312 and Ta_54145, the amino acid that corresponds to Ser143 in TaLcc1 is replaced by Gly and in Tv_48916, by Ala. Additionally, in Th_539081, the residue that corresponds to Ser511 in TaLcc1 is replaced by a Thr (Fig. 2). This result suggests that *Trichoderma* laccases have adopted various strategies to facilitate the transfer of protons to the trinuclear site, thus modifying its catalytic activity.

An essential aspect of the catalytic activity of laccases is the mode of interaction and reaction with various substrates. The availability of a chemical compound to be used as a laccase substrate depends on both the nature and position of the substituents in the phenolic ring of the compound and also on the chemical environment of the sustrate binding site. The amino acid residues that constitute the substrate cavity form loops and are founds in domains II and III. In the laccases of various organisms, the loops have different amino acid compositions, which results in diversity in the size and shape of the substrate binding site [43]. The nine *Trichoderma* spp. proteins present the substrate binding sites that are described in the tridimensional structure of crystallized laccases. As in other laccases, the sequences that form loops I-IV are little conserved in comparison with MaL and TaLcc1 (wild proteins of *M. albomyces* and *T. arenaria*, respectively), except in loop IV in C7–C8, which is also part of the L4 segment (Fig. 2). It has been determined that Pro192 in MaL (Ala193 in TaLcc1) in the loop I/B1–B2 interacts with the organic substrate [31]. This residue is present in five *Trichoderma* spp. laccases, although in this position, Th_539081 and Tv_194054 possess a Gln residue whereas Tas_68620 and Tas_71665 have Thr and Ser, respectively (Fig. 2). In B4–B5, fungal laccases typically have Glu or Asp as the substrate ligand [31], [42]. Directed mutagenesis studies performed on MaL have shown that the Glu235 (Asp 236 in TaLcc1) carboxy group is of great importance for substrate binding because it stabilizes the cationic radical that is formed when Hys508 initiates the catalytic cycle [44]. However, in Tas_71665, Tr_122948 and Tv_194054, this residue is replaced by Ala. The multiple alignment of these sequences with other members of the MCO family revealed that in the same position, Ala is only present in a laccase of the dermatophyte fungus *A. gypseum* (anamorph = *Microsporum gypseum*). In the recombinant *M. albomyces* laccase (rMaL) expressed in *T. reesei*, the Cys residue of the tripeptide Ala297-Cys298-Gly299 (Leu297-Cys298-Gly299 in TaLcc1) located in loop II/B7-B8 is conserved in *Trichoderma* spp. laccases. This amino acid is also involved in substrate binding and was found in *N. crassa, P. anserina* and *C. parasitica* laccases [44]. Furthermore, the involvement of rMaL Phe427 (Val428 in TaLcc1) of loop IV/C4–C5 in aligning substrate molecules in the correct orientation for oxidation has been suggested [44]. In the laccases of various organisms, including *Trichoderma* spp., this residue varies considerably, although the majority of basidiomycete laccases display Pro in this position. The differences found in the loop sequences of *Trichoderma* spp. laccases suggest a low substrate specificity and, most likely, various catalytic capacities, which indicates that each possesses different physiological functions, which is reinforced by subcellular localization (see below).

A preserved segment of four residues of amino acids has been identified at the C-terminal end of *Trichoderma* spp. laccases (Fig. 2), a highly conserved sequence in ascomycetes corresponding to the consensus Asp-Ser-Gly-(Leu/Ile/Val). In laccases of the ascomycetes *M. albomyces* [31], *P. anserina* [35], *T. arenaria* [42], *N. crassa* [45] and *M. thermophila* [46] the C-terminal end is post-translationally processed, leaving the active protein with the sequence Asp-Ser-Gly-Leu (DSGL) as the final amino acid residues at this end. As in the *Trichoderma* spp. laccases, the majority of ascomycete laccases do not present this C-terminal extension, which is removed post-translationally. Determination of the three-dimensional structure of MaL and TaLcc1 revealed that the C-terminal end DSGL is packed within a tunnel that leads to the trinuclear site and forms a plug [31]. In the crystal structure of other known laccases, this cavity is open and allows molecular oxygen to access the catalytic site [42]. In MaL and TaLcc1, the C-terminus plug impedes the movement of molecular oxygen and other solvents to the enzyme. Furthermore, the C-terminus carboxyl group forms a hydrogen bridge with the lateral chain of Hys140 in MaL (Hys141 in TaLcc1), which also coordinates copper T2. Directed mutagenesis studies performed on MaL cDNA revealed that a change or deletion of the C-terminal end dramatically affects enzyme activity [47]. Given those findings, it was suggested that ascomycete laccases use the C-terminal DSGL plug to carry out their catalytic function, forming a proton transfer pathway [42]. The C-terminus block appears to be a characteristic trait of ascomycete laccases because it has not been described in basidiomycete laccases. Nevertheless, *Rigidopurus lignosus* R1L laccase presents the C-terminus sequence DSGLA. Among basidiomycete laccases, R1L is the most closely related phylogenetically with ascomycete laccases; for this reason, it was initially suggested that this sequence of the C-terminal end was more an evolutionary relic than a functional characteristic of the enzyme [39]. However, more recent observations documented in this study have shown that the C-terminal end DSGL is not an evolutionary relic in fungi, as it provides important functions for the ascomycete enzyme. Furthermore, even when laccases of basidiomycetes lacks DSGL motif, it was recently established −using directed evolution approach − that the C-terminus would play a role in enzyme performance by influencing optimal pH and *Km* values for phenolic compounds [48]. This evidence requires further comparative studies between ascomycetes and basidiomycetes regarding the evolution of the C-terminal end and its functional role.

In the course of the analysis to identify laccases *sensu stricto* in *Trichoderma* and to compare them with those reported in other ascomycetes, the particular case of the 6 enzymes reported for *Aspergillus niger* [8] emerged, specifically those designated as Mco G, Mco J and Mco M. When we perform an analysis to find the signatures of laccases *sensu stricto* (Table 1), we find that the Mco G protein lacks the DSGL motif. Furthermore, Mco J and Mco M have a deletion of approximately 50 aa immediately after the L4 segment, which includes the DSGL motif. Interestingly, it was found that these two last “truncated” enzymes oxidize a limited number of substrates, whereas the former attacks all probed substrates. This suggests that some laccases from ascomycetes can be partially functional even if not presenting all the elements to be considered as laccases *sensu stricto*. These beings a special case and beyond the objectives of the present work, further analyses of these three enzymes were not performed and they were excluded from the phylogenetic analysis (see below).

Over the course of the evolutionary process, laccases have maintained a high degree of similarity in terms of amino acid sequence and three-dimensional structure. Generally, the laccase sequences of members of a group of fungi exhibit levels of amino acid identity of 50% or more, whereas the identity levels between sequences of members of different groups is approximately 30% [49]. These identity values are met, in general, for *Trichoderma* spp. laccases. The identity percentage among the 9 laccases varied between 30 and 88% (Table 3). The Ta_54145/Tas_514312/Tv_48916 triad is more similar among themselves with an identity value above 83%. The rest of the laccases possess identity values between 51 and 86%. An identity percentage comparison between laccases of a single species has not been conducted in ascomycetes. In the case of basidiomycetes, the percentages of identity of the 8 *C. cinerea* laccases varied between 46 and 77% [50], whereas in the case of *P. ostreatus,* these values were between 45 and 89% between the 6 enzymes found [49]. Thus, *Trichoderma* laccases present a greater interval of identity than those found in basidiomycete species, indicating a strong selective pressure on the various genes.

**Table 3:** Identity (%) among protein sequences of the members of the *Trichoderma* laccase family.

	Protein	2	3	4	5	6	7	8	9
**1**	**Tas_68620**	85.4	76.9	77.7	77.1	53.1	31.7	32.1	30.9
**2**	**Ta_40409**		75.2	76.1	76.1	53.1	33.6	33.4	32.7
**3**	**Th_539081**			86.7	75.7	52.1	30.5	31.5	30.5
**4**	**Tv_194054**				77.3	51.9	32.1	32.4	31.0
**5**	**Tr_122948**					51.0	30.6	31.0	30.8
**6**	**Tas_71665**						30.9	31.2	30.4
**7**	**Tas_154312**							88.5	86.2
**8**	**Ta_54145**								83.7
**9**	**Tv_48916**								

Proteins designated as the corresponding genes in the JGI GeneBank.

With respect to their relationship to the laccases of other fungal species, Tas_154312, Ta_54145 and Tv_48916 proteins share 53% identity with *G. graminis var. tritici* and *G. graminis var. graminis* Lac2 [33] and an identity of between 63 and 65% with *F. oxysporum* Lcc4 laccase [10]. The group of enzymes Tas_68630, Ta_40409, Th_539081, Tr_122948 and Tv_194054 possess an identity greater than 63% with *F. oxysporum* lacc1 [10] and less than 30% with other ascomycete laccases. Interestingly, *F. oxysporum lcc1* laccase is an intracellular laccase like the *Trichoderma* laccases, which show a stronger phylogenetic relation with it (see below). The Tas_71665 protein has an identity of between 51.4 and 56.2% with the putative laccases of *B. fuckeliana*, *S. sclerotiorum* and *P. tritici-repentis*. The identities of the nine *Trichoderma* spp. laccases with respect to crystallized MaL and TaLcc1 laccases are between 27 and 37%. When comparing *Trichoderma* laccases with those from the basidiomycete fungi *T. versicolor*, *P. ostreatus* and *A. bisporus*, the identity is below 25%. The identity values clearly show the separation of the *Trichoderma* spp. laccases into three subfamilies, which is directly related to the putative subcellular localization of laccases *sensu stricto* (see following paragraph).

### Prediction of Subcellular Localization of *Trichoderma* spp. Laccases and Possible Physiological Functions

The majority of known fungal laccases are monomeric proteins with extracellular activity, although intracellular laccases have also been identified, particularly in white rot fungi [3]. The localization of laccases is associated with their physiological function and determines the range of substrates available for the enzyme. In fungi, the functions of extracellular laccases related to the degradation of lignocellulose material, recycling of organic material, reduction of oxidative stress and pathogenesis toward plants and animals have been extensively studied [3], [4]. Of the nine *Trichoderma* spp. laccases, it was determined that four correspond to extracellular laccases: one each in *T. atroviride* and *T. virens,* and two in *T. asperellum.* The rest are intracellular proteins found in the five species analyzed (Table 2). The putative signal peptide of the extracellular laccases corresponds to the first 18 residues and presents the typical characteristics of signal peptides, that is, a highly hydrophobic region and Ala and Val residues in positions -1 and -3, respectively, relative to the cleavage site [51]. The mature forms of the laccases Tas_71665, Tas_154312, Ta_54145 and Tv_48916 possess between 9 and 11 putative N-glycosylation sites (Table 2). The average glycosylation is usually between 10 and 25%, although laccases with a carbohydrate content of greater than 30% have been detected [2]. Glycosylation influences enzyme secretion, and it has been suggested to play an important role in catalytic center stabilization, protection against hydrolysis, copper retention, and laccase thermal stability [52]. Extracellular laccase activity has previously been reported in *Trichoderma* spp. [18], [21], arriving in certain cases at purification of the protein [23], [24]. Levasseur *et al.* [25] reported homologous overexpression of the *T. reesei* gene 124079 in the strain Rut-C30, and even when the recombinant protein presented biochemical properties similar to those reported for other laccases, the structural analysis carried out in this study suggests that the enzyme TrLAC1 studied by those authors corresponds to a pigment synthesis MCO and not to a laccase in *sensu stricto*. That is, the amino acid sequence obtained for this enzyme does not present either the SDS-gate or the C-terminal end DSGL/I/V. Recently, Catalano *et al.* [26] evaluated the participation of the extracellular *T. virens* LCC1 laccase (corresponding to laccase Tv_48916 analyzed in this study) in mycoparasitism towards sclerotia of the phytopathogens *B. cinerea* and *S. sclerotiorum*. The hypothesis in that study was that LCC1 is capable of attacking the sclerotia melanin of the studied fungi. However, although their results show that the enzyme can participate in mycoparasitism by *T. virens* against *S. sclerotiorum*, the same result was not obtained against *B. cinerea* sclerotia, making it necessary to conduct more studies in this area. It would also be important to experimentally evaluate other functions of extracellular *Trichoderma* laccases, such as those cited at the beginning of this section.

The analysis carried out with various bioinformatic packages suggests the intracellular localization of laccases Ta_40409, Tr_122948, Tv_194054, Th_539081 and Tas_68620. Unlike extracellular laccases, little is known about the activity of intracellular laccases, both for the genus *Trichoderma* and for other fungal species. In several reports, the presence of laccase activity associated with the membrane or periplasmic space during the maturation of *T. atroviride, T. viride* and *T. harzianum* conidia [21], [22] has been suggested. The results of the present study show that *Trichoderma* spp. do not possess laccases associated with the plasma membrane, such laccase activity associated with conidia reported in those studies is possibly due to the extracellular enzyme that remained trapped in the periplasmic space or the cell wall during the process of maturation of these structures. In this sense, fungal proteins have been reported to be located either adhering to the cell wall or in the extracellular medium, among which are included hydrophobins and adhesins [53], [54], as well as hydrolytic enzymes [55]. Although, in certain cases, the interaction of the protein with the cell wall is only transitory, occurring while the protein reaches the extracellular medium, in other cases, specific mechanisms have been proposed for the retention of the proteins in the wall and their simultaneous localization in the extracellular medium [56]. There has also been documentation of *A.oryzae* enzymes that are secreted into the medium when the fungus is grown in a solid substrate but are retained in the cell wall when the fungus grows in submerged cultures [57]. This finding indicates that it is possible that some *Trichoderma* laccases remain trapped in the periplasmic space or cell wall during conidia maturation, which is a possibility that should be investigated more thoroughly in the future. In ascomycetes, the activity of intracellular laccase has been detected in *H. acidophila* and has been related to the synthesis of melanin [7]. In the human pathogenic fungus *Cryptotoccus neoformans,* laccase activity is found to be associated with the membrane and constitutes a virulence factor [56], whereas the phytopathogenic fungus *F. oxysporum* possesses two intracellular laccases, Lcc1 and Lcc3, which may be involved in the protection of the fungus against oxidative stress and toxic compounds [10]. In the basidiomycetes *L. bicolor* [11] and *P. ostreatus* [15], these isoenzymes are involved in the development of the fruiting body. In addition, it is possible that the intracellular laccases of fungi participate in the transformation of low molecular weight phenolic compounds produced in the cell [3]. Laccases associated with conidia are linked to the synthesis of pigments and other substances that protect the cell from stress factors, such as enzymatic lysis, temperature and UV light [3], [10]. It is possible that *Trichoderma* intracellular laccases are related to any of the processes described above; further experimental work is needed to confirm any of these functions.

Surprisingly, when using various bioinformatics programs to analyze four of the laccases classified as intracellular by the package SignalP 4.0, the laccases presented a signal peptide and processing characteristics of mitochondrial localization (Fig. 2; Table 4). The MitoProt program shows processing sites congruent with the mitochondrially located peptide of the other packages (Table 4). These results should be viewed cautiously, as currently there are no reports of mitochondrial localization of laccases either in fungi or in any other biological group in which this enzymatic activity has been found.

**Table 4:** Signal peptide sequences from *Trichoderma* laccases predicted by different software programs.

Protein	SignalP^a^	PrediSi^a^	TargetP^b^	iPSORT^c^	MitoProt^d^
**Tas_68620**	N(0.140)	N (0.4239)	M (0.776)	–	CleavSite 14 (0.3382)
**Ta_40409**	N(0.152)	N (0.373)	M (0.676)	mTP (0.166667)	CleavSite 4 (0.746)
**Th_539081**	N(0.168)	N (0.0)	M (0.931)	mTP (0.133333)	– (0.3726)
**Tv_194054**	N(0.159)	N (0.4244)	M (0.898)	mTP (0.166667)	CleavSite 13 (0.9523)
**Tr_122948**	N(0.148)	N (0.0)	Other (0.779)	–	– (0.1994)
**Tas_71665**	Y(0.680)	Y (0.5662)	S (0.905)	SP (2.06667)	– (0.329)
**Tas_154312**	Y(0.878)	Y (0.7464)	S (0.887)	SP (2.12)	CleavSite 26 (0.2047)
**Ta_54145**	Y(0.827)	Y (0.7953)	S (0.863)	SP (1.72667)	– (0.45)
**Tv_48916**	Y(0.803)	Y (0.6639)	S (0.843)	SP (1.48667)	– (0.1851)

The values between parentheses are the probability that the prediction is accurate. ^a^N, non present; Y, present. ^b^M, mitochondrial; S, secretory. ^c^mTP, mitochondrial targeting peptide; SP, secretory signal peptide. ^d^CleavSite, location of signal peptide cleavage sites.

With the unexpected finding of signs mitochondrial localization of laccase in *Trichoderma*, the first question that arises is whether this is feasible for enzymes that in fungi have only been reported as either cytoplasmic or associated with the plasma membrane or extracellular. Possible hypotheses, assuming that the prediction of mitochondrial localization is correct, arise from what has been documented for proteins in other fungi. There are examples in fungi of subcellular localization changes or the presence in two distinct cellular compartments of the same enzymatic activity. The latter case has been referred to as dual localization, dual targeting or dual distribution [58]. In *S. cerevisiae,* it has been found that up to a third of the proteins considered to be mitochondrial can present an alternative subcellular localization [59]. Among the best-studied examples of dual mitochondria-cytoplasmic localization in fungi are aconitase [60] and fumarase [61]. Moreover, in these two examples, the proteins present in mitochondria and cytoplasm come from the same gene, which generates a single transcript and a single translation product, that is, they are not isoenzymes in distinct compartments. However, in these two examples, the “original” localization of the protein is mitochondrial, and the “new” localization is cytoplasmic, which is the opposite of what would be occurring with the products of *Trichoderma* laccase proteins. Nevertheless, it is possible to consider the relocalization of a secretion protein to mitochondria in fungi. Recently it has been documented that tryptophan-rich sensory protein/peripheral-type benzodiazepine receptor (referred to as TspO/MBR) that is found in the Golgi-associated secretory pathway in plants is directed toward mitochondria when expressed heterologously in *S. cerevisiae* [62]. The modification of the subcellular localization of a protein may be caused by a change of a single amino acid in the signal peptide, which can be generated by a single nucleotide mutation [63].

The second question that arises pertains to the function of a laccase in mitochondria. Experimental evidence suggests that a protein that reaches a new subcellular location can develop new functions [63]. Although it is difficult to speculate about the possible function of a mitochondrial laccase in fungi, it is feasible to establish hypotheses based on our understanding of the structural aspects of this enzyme. As mentioned above, laccases are enzymes that can bind four copper atoms, which are important for catalytic activity. Intracellular Cu^+2^ can have damaging effects because it induces the formation of reactive oxygen species (ROS); therefore, there are mechanisms that regulate its concentration [64]. Further, it has been documented that mitochondria possess a pool of copper that responds to changes in copper levels in the cytoplasm [65]. It is possible that the mitochondrial location of *Trichoderma* laccases contributes to the homeostasis of mitochondrial Cu^+2^ under particular circumstances, which is a possibility that would be important to evaluate experimentally in the future.

The prediction of the subcellular localization of eukaryotic proteins is a complicated task in that there is always a certain degree of uncertainty; therefore, protocols and bioinformatics packages have been designed that have optimized the certainty of the prediction [66]. Although speculative hypotheses are proposed in agreement with bioinformatics and experimental evidence collected in eukaryotes in general and in fungi in particular, it would be most prudent to assume that the four *Trichoderma* enzymes can be considered to be intracellular, which is supported by the phylogenetic analysis (see following paragraph). The here described role of putative mitochondrial signal peptides in the subcellular localization of *Trichoderma* laccases can be experimentally verified in the future. One way to do this, is to design an expression vector in which the putative mitochondrial target sequences are fused to the green fluorescent protein (gfp) gene and monitoring the localization of the expressed recombinant protein.

### Phylogenetic Analysis of *Trichoderma* spp. Laccases

**Figure 3:**
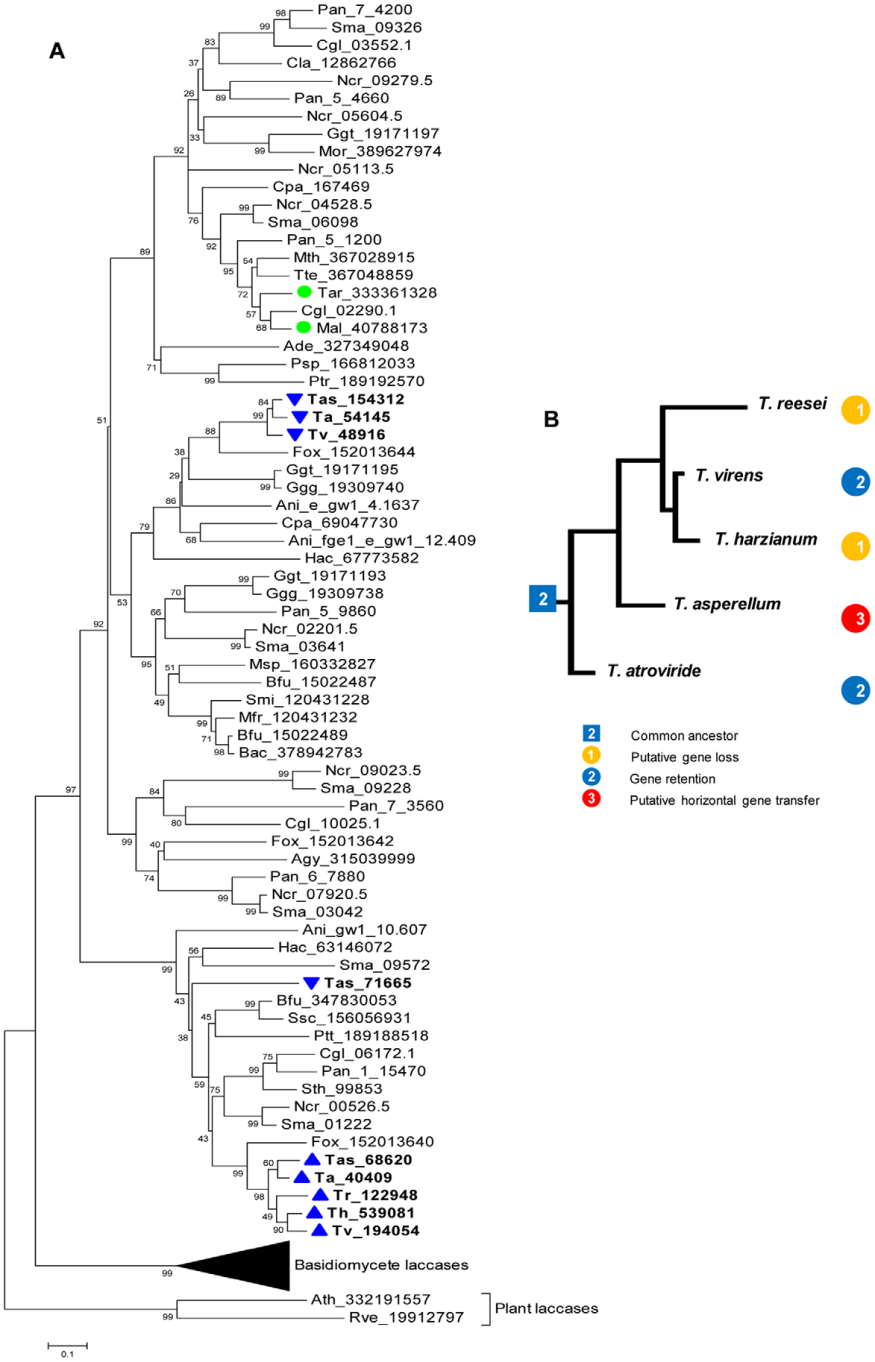
Phylogenetic analysis of laccases and of *Trichoderma* species. (**A**) Neighbor-joining tree of the deduced amino acid sequences of the five *Trichoderma* laccases and selected laccases of other ascomycetes, basidiomycetes and plants**.** The tree is calculated using the Jones-Taylor-Thornton (JTT) model in Mega Ver 5.05 based on a ClustalX alignment. Bootstrap values are from 1000 replications. The scale bar indicates a distance equivalent to 0.2 amino acid substitutions per site. Species and strains are indicated in the experimental procedures. Upward triangles indicate *Trichoderma* intacellular laccases and downward triangles extracellular ones. Green circles denote the laccases from *M. albomyces* [44] and *T. arenaria* [42]. (**B**) Phylogenetic tree of *Trichoderma* species showing gain, loss and retention of laccase genes.

A phylogenetic analysis performed according to two distinct criteria separated *Trichoderma* laccases into two distinct clades with a bootstrap value of 99%. Interestingly, all intracellular laccases are grouped in the first of these clades. The remaining three extracellular laccases are included in the second clade with the exception of Tas_71665 (Fig. 3a), which grouped in the clade of intracellular proteins but in a different terminal branch.

Although *Trichoderma* extracellular laccases exhibit close phylogenetic relationships with *F. oxysporum*, *G. gramminis*, *A. niger* and *C. parasitica* orthologs, intracellular laccases show relationships with *S. thermophile*, *N. crassa*, *S. macrospora, P. anserina, F. oxysporum* and *C. globosum* orthologs. One may suggest the hypothesis that the phylogenetic closeness between these isoenzymes involves structural similarities in terms of the regions and amino acids discussed above for *Trichoderma* laccases. It is important to emphasize that all the proteins included in the phylogenetic analysis of Figure 3a were “curated” according to the same criteria as those of *Trichoderma* (Table 1) to ensure that they included only laccases *sensu stricto*. Because of this approach, the phylogram generated here excludes proteins that were used in previous phylogenetic analyses of ascomycete laccases [12], [25], which are possibly another members of MCO family. In the future, it will be important to consider this aspect of treatment to obtain more robust phylogenetic patterns that will provide a clearer idea of the evolutionary process of this enzymatic function in ascomycetes. This approach will allow for a better definition of laccases with similar functions among distinct species of this group of fungi.

The phylogenetic separation of the subfamilies of laccases is fully congruent with the analysis of sites or characteristic sequences of each subfamily of genes discussed above. Both the structure and number of genes found in the species of *Trichoderma* and the phylogenetic analysis suggest that the common ancestor of said genus possessed two laccases, with one being intracellular and the other extracellular.

Comparative genomic analysis together with the results of molecular phylogeny leads to the conclusion that the ancestral state of *Trichoderma*/*Hypocrea* was mycoparasitical and that it later acquired saprophytic characteristics that helped it to pursue its prey through lignified substrates [27]. These studies have shown that *T. atroviride* is the earliest species within the genus, whereas *T. virens* and *T. reesei* appeared later. Furthermore, during the evolutionary process, *T. reesei* lost a significant quantity of the genetic information present in *T. atroviride*, which was retained in *T. virens* [27]. The number of *Trichoderma* laccase genes and the phylogenetic analysis of the proteins coded by these genes are congruent with the described scenario (Fig. 3b). With the generated data, it is possible to support the hypothesis that during the process of speciation, species such as *T. atroviride* and *T. virens* maintained the original number of laccase genes present in the common ancestor, while *T. harzianum* and *T. ressei* represent species that lost copies of extracellular laccase. On the other hand, several lines of evidence strongly suggest that the Tas_71665 gene of *T. asperellum* was acquired through a horizontal gene transfer event. This gene has the following structural features, which are discussed in the previous sections: i) it has the lowest GC-content (46%) of all known laccase genes; ii) it has a unique structure with seven introns; iii) the genome context of this gene differs from those of other laccase genes in *T. asperellum*; iv) no orthologous genes were found in the other analyzed *Trichoderma* species; v) compared to laccases in other genera of ascomycetes, this protein shows higher identity values (51.4–52.6%) with laccases of *B. fuckeliana*, *S. sclerotiorum* and *P. tritici-repentis*; vi) in the phylogenetic analysis, the protein encoded by this gene does not cluster toghether with other laccases from *Trichoderma*. Most of these features have been recognized as evidence of horizontal gene transfer in fungi [67]. It has been previously suggested that in *S. macrospora* and *P. anserina* –belonging to the same Order as *Trichoderma* (Sordariales) – the acquisition of laccase genes occured through horizontal transference – [12]; the author postulating the possibility of gene transfer from *S. sclerotiorum* to *S. macrospora.* Taken together, our results strongly suggest that *T. asperellum* acquired the Tas_71665 gene through horizontal gene transfer, either from one of the known necrotrophic phytopathogens *B. fuckeliana*, *S. sclerotiorum* and *P. tritici-repentis*, or from a related fungus. This is noteworthy because there is experimental evidence showing mycoparasitic activity of *Trichoderma*, althought not specifically *T. asperellum*, against *B. fuckeliana* and *S. sclerotiorum* [26], which indicates that this gene could have been acquired during the mycoparasitic process. This pattern of retention/loss/gain of laccase genes by groups of species in *Trichoderma* may reflect phenomena of selective pressure associated with the lifestyle of each of these species.

The phylogeny obtained shows that the hypothesis of two laccase genes in the common *Trichoderma* ancestor with later events of retention, loss or gain can be extended to the Ascomycetes group. In fact, this asymmetrical pattern of change in gene families has been reported for the case of family 28 of ascomycetes glycosyl hydrolases [68], where duplications and losses of genes in distinct groups of fungi within this subdivision are observed. Detailed studies of ascomycete genomes to locate more laccase isoenzymes and carry out a broader phylogenetic analysis would allow for the corroboration of this hypothesis.

Analysis of the evolutionary patterns of *Trichoderma* laccases shows that the proportion of synonymous substitutions of the majority of enzymes varies between 56 and 78%, indicating saturation (Table 5). The exceptions were Tas_154312 and Ta_54145 (36%), as well as Tas_68620 and Ta_40409 (43%). The majority of the non-synonymous substitutions show values higher than 70%, indicating saturation. A low percentage of non-synonymous substitution is found in 36–37%, between 6 and 8%. These values, together with the percentage non-synonymous/percentage synonymous (pn/ps) proportions, show that the majority of *Trichoderma* extracellular laccases evolved under a process of purifying selection with the exception of Ta_71665, which has values that indicate neutral modifications (Table 5). In the case of the intracellular laccases, the majority of values indicate neutral changes, although in the case of the pair Tas_68620 and Ta_40409, purifying selection is shown.

**Table 5:** Synonymous and non-synonymous substitution rates (%) between lacccase genes of *Trichoderma* spp.

Gene	Value^a^	Ta_54145	Tv_48916	Tas_71665	Th_539081	Tv_194054	Tr_122948	Tas_68620	Ta_40409
**Tas_154312**	**ps**	36	60	78	74	77	71	76	72
	**pn**	6	7	75	72	73	70	74	74
	**pn/ps**	0.16	0.11	0.96	0.97	0.94	0.98	0.97	1.02
**Ta_54145**	**ps**		56	78	72	74	68	77	76
	**pn**		8	75	73	73	69	75	75
	**pn/ps**		0.14	0.96	1.01	0.98	1.01	0.97	0.98
**Tv_48916**	**ps**			76	75	74	70	74	72
	**pn**			74	73	73	71	74	74
	**pn/ps**			0.97	0.97	0.98	1.01	1.00	1.02
**Tas_71665**	**ps**				76	77	74	74	74
	**pn**				75	70	73	74	74
	**pn/ps**				0.98	0.9	0.98	1.00	1.00
**Th_539081**	**ps**					75	72	66	71
	**pn**					72	72	36	37
	**pn/ps**					0.96	1.00	0.54	0.52
**Tv_194054**	**ps**						73	69	69
	**pn**						72	71	71
	**pn/ps**						0.98	1.02	1.02
**Tr_122948**	**ps**							68	66
	**pn**							72	72
	**pn/ps**							1.05	1.09
**Tas_68620**	**ps**								43
	**pn**								8
	**pn/ps**								0.18

Genes designed as in the JGI GeneBank. ^a^ps, percentage of synonymous substitutions; pn, percentage of non-synonymous substitutions; pn/ps, ratio of synonymous, non synonymous substitutions.

Levasseur *et al*. [25] found that the TrLACgene, identified by those authors as a *T. reesei* laccase, evolved under positive selection. However, as mentioned above, our data suggest that this gene is not a laccase *sensu stricto* but rather is another member of the MCO family. To this date, there are no studies that would allow us to compare the evolutionary patterns of *Trichoderma* laccases found in this study with those of ascomycete laccases.

### Conclusions

The search for laccases *sensu stricto* in ascomycetes involves the location in the amino acid sequence of motifs that are not present in basidiomycete laccases. The characterization of such motifs allows for better description of the possible functional properties of the proteins. The identification of ascomycete laccases *sensu stricto* is important to conduct robust phylogenetic analyses of this enzymatic function. The genus *Trichoderma* has preserved a limited number of functional laccase genes in the course of the evolutionary process. Structural and phylogenetic evidence suggests that the common ancestor of *Trichoderma* spp. had two laccase genes, with one being intracellular and the other extracellular. Species within the genus *Trichoderma* tend to preserve intracellular laccase activity, whereas evolution patterns of extracellular activity are variable. In the case of *T. asperellum*, there is strong evidence of horizontal gene transference through the mycoparasitic proceses. The herein presented data will contribute to the understanding of the functional role of laccases in the genus *Trichoderma* and to the optimization of their biotechnological applications.

## References

[1] Quintanar L, Stoj C, Taylor AB, Hart PJ, Kosman DJ, et al (2007) Shall we dance? How a multicopper oxidase chooses its electron transfer partner. Acc Chem Res 40: 445–452. 17425282 10.1021/ar600051a

[2] Giardiana P, Faraco V, Pezzella C, Piscitelli A, Vanhulle S, et al (2010) Laccases: a never-ending story. Cell Mol Life Sci 67: 369–385. 19844659 10.1007/s00018-009-0169-1 PMC11115910

[3] Baldrian P (2006) Fungal laccases – occurrence and properties. FEMS Microbiol Rev 30: 215–242. 16472305 10.1111/j.1574-4976.2005.00010.x

[4] Schouten A, Wagemakers L, Stefanato FL, van der Kaaij RM, van Kan JAL (2002) Resveratrol acts as a natural profungicide and induces self-intoxication by a specific laccase. Mol Microbiol 43: 883–894. 11929539 10.1046/j.1365-2958.2002.02801.x

[5] Maté D, García-Ruíz E, Camarero S, Alcalde M (2011) Directed evolution of fungal laccases. Curr Genomics 12: 113–122. 21966249 10.2174/138920211795564322 PMC3129045

[6] Shraddha, Shekher R, Sehgal S, Kamthania M, Kumar A (2011) Laccase: microbial sources, production, purification, and potential biotechnological applications. Enzyme Res doi:10.4061/2011/217861. 10.4061/2011/217861 PMC3132468 21755038

[7] Tetsch L, Bend J, Hölker U (2006) Molecular and enzymatic characterization of extra- and intracellular laccases from the acidophilic ascomycete *Hortaea acidophila* . Anton Leeuw 90: 183–194. 10.1007/s10482-006-9064-z 16871425

[8] Tamayo-Ramos JA, Barends S, Verhaert RMD, de Graaff LH (2011) The *Aspergillus niger* multicopper oxidase family: analysis and overexpression of laccase-like encoding genes. Microb Cell Fact 10: 78. 21981827 10.1186/1475-2859-10-78 PMC3200161

[9] Hoshida H, Nakao M, Kanazawa H, Kubo K, Hakukawa T, et al (2001) Isolation of five laccase gene sequences from the white-rot fungus *Trametes sanguinea* by PCR, and cloning, characterization and expression of the laccase cDNA in yeast. J Biosci Bioeng 92: 372–380. 16233113 10.1263/jbb.92.372

[10] Cordoba CD, Roncero MIG (2008) Functional analyses of laccase genes from *Fusarium oxysporum* . Mycology 98: 509–518. 10.1094/PHYTO-98-5-0509 18943218

[11] Courty PE, Hoegger PJ, Kilaru S, Kohler A, Buée M, et al (2009) Phylogenetic analysis, genomic organization, and expression analysis of multi-copper oxidases in the ectomycorrhizal basidiomycete *Laccaria bicolor* . New Phytol 182: 736–750. 19243515 10.1111/j.1469-8137.2009.02774.x

[12] Pöggeler S (2011) Evolution of multicopper oxidase genes in coprophilous and non-coprophilous members of the order sordariales. Curr Genomics 12: 95–103. 21966247 10.2174/138920211795564368 PMC3129052

[13] Hoegger PJ, Kilaru S, James TY, Thacker JR, Kües U (2006) Phylogenetic comparison and classification of laccase and related multicopper oxidase protein sequences. FEBS J 273: 2308–2326. 16650005 10.1111/j.1742-4658.2006.05247.x

[14] Kilaru S, Hoegger PJ, Kües U (2006) The laccase multi-gene family in *Coprinopsis cinerea* has seventeen different members that divide into two distinct subfamilies. Curr Genet 50: 45–60. 16775746 10.1007/s00294-006-0074-1

[15] Lettera V, Piscitelli A, Leo G, Birolo L, Pezzella C, et al (2010) Identification of a new member of *Pleurotus ostreatus* laccase family from mature fruiting body. Fungal Biol 114: 724–730. 20943181 10.1016/j.funbio.2010.06.004

[16] Kubicek CP, Bissett J, Druzhinina I, Kullnig-Gradinger C, Szakacs G (2003) Genetic and metabolic diversity of *Trichoderma*: a case study on South-East Asian isolates. Fungal Genet Biol 38: 310–319. 12684020 10.1016/s1087-1845(02)00583-2

[17] Schuster A, Schmoll M (2010) Biology and biotechnology of *Trichoderma* . Appl Microbiol Biotechnol 87: 787–799. 20461510 10.1007/s00253-010-2632-1 PMC2886115

[18] Assavanig A, Amornkitticharoen B, Ekpaisal N, Meevootisom V, Flegel TW (1992) Isolation, characterization and function of laccase from *Trichoderma* . Appl Microbiol Biotechnol 38: 198–202.

[19] Krastanov AI, Gochev VK, Girova TD (2007) Nutritive medium dependent biosynthesis of extracellular laccase from *Trichoderma* spp. Bulg J Agric Sci 13: 349–355.

[20] Gochev VK, Krastanov AI (2007) Isolation of laccase producing *Trichoderma* spp. Bulg J Agric Sci 13: 171–176.

[21] Hölker H, Dohse J, Höfer M (2002) Extracellular laccases in ascomycetes *Trichoderma atroviride* and *Trichoderma harzianum* . Folia Microbiol 47: 423–427. 12422522 10.1007/BF02818702

[22] Pokorný R, Vargovič P, Hölker U, Janssen M, Bend J, et al (2005) Developmental changes in *Trichoderma viride* enzymes abundant in conidia and the light-induced conidiation signalling pathway. J Basic Microbiol 45: 219–229. 15900543 10.1002/jobm.200410354

[23] Sadhasivam S, Savitha S, Swwaminathan K (2009) Redox-mediated decolorization of recalcitrant textile dyes by *Trichoderma harzianum* WL1 laccase. World J Microbiol Biotechnol 25: 1733–1741.

[24] Chakroun H, Mechichi T, Martinez MJ, Dhouib A, Sayadi S (2010) *Trichoderma atroviride*: application on bioremediation of phenolic compounds. Process Biochem 45: 507–513.

[25] Levasseur A, Saloheimo M, Navarro D, Andberg M, Pontarotti P, et al (2010) Exploring laccase-like multicopper oxidase genes from the ascomycete *Trichoderma reesei*: a functional, phylogenetic and evolutionary study. BMC Biochem 11: 32. 20735824 10.1186/1471-2091-11-32 PMC2939539

[26] Catalano V, Vergara M, Hauzenberger JR, Seiboth B, Sarrocco S, et al (2011) Use a non-homologous end-joining-deficient strain (delta-ku70) of the biocontrol fungus *Trichoderma virens* to investigate the function of the laccase gene lcc1 in sclerotia degradation. Curr Genet 57: 13–23. 20872221 10.1007/s00294-010-0322-2 PMC3023040

[27] Kubicek CP, Herrera-Estrella A, Seidl-Seiboth V, Martínez DA, Druzhinina IS, et al (2011) Comparative genome sequence analysis underscores mycoparasitism as the ancestral life style for *Trichoderma* . Genome Biol 12: R40. 21501500 10.1186/gb-2011-12-4-r40 PMC3218866

[28] Martínez D, Berka RM, Henrissat B, Saloheimo M, Arvas M, et al (2008) Genome sequencing and analysis of the biomass-degrading fungus *Trichoderma reesei* (syn. *Hypocrea jecorina*). Nat Biotechnol 26: 553–560. 18454138 10.1038/nbt1403

[29] Kumar S, Phale P, Durani S, Wangikar PP (2003) Combined sequence and structure analysis of the fungal laccase family. Biotechnol Bioeng 83: 386–394. 12800133 10.1002/bit.10681

[30] Hakulinen N, Andberg M, Kallio J, Koivula A, Kruus K, et al (2008) A near atomic resolution structure of a *Melanocarpus albomyces* laccase. J Struct Biol 162: 29–39. 18249560 10.1016/j.jsb.2007.12.003

[31] Hakulinen N, Kiiskinen LL, Kruus K, Saloheimo M, Paananen A, et al (2002) Crystal structure of a laccase from *Melanocarpus albomyces* with an intact trinuclear copper site. Nature Struct Biol 9: 601–605. 12118243 10.1038/nsb823

[32] Eggert C, LaFayette PR, Temp U, Eriksson KEL, Dean JFD (1998) Molecular analysis of a laccase gene from the white rot fungus *Pycnoporus cinnabarinus* . Appl Environ Microbiol 64: 1766–1772. 9572949 10.1128/aem.64.5.1766-1772.1998 PMC106228

[33] Litvintseva AP, Henson JM (2002) Cloning, characterization, and transcription of three laccase genes from *Gaeumannomyces graminis* var. *tritici*, the take-all fungus. Appl Environ Microbiol 68: 1305–1311. 11872481 10.1128/AEM.68.3.1305-1311.2002 PMC123725

[34] Martin C, Pecyna M, Kellner H, Jehmlich N, Junghanns C, et al (2007) Purification and biochemical characterization of a laccase from the aquatic fungus *Myrioconium* sp. UHH 1–13–18–4 and molecular analysis of the laccase-encoding gene. Appl Microbiol Biotechnol 77: 613–624. 17955194 10.1007/s00253-007-1207-2

[35] Fernández LJ, Stahl U (1996) Isolation and characterization of a laccase gene from *Podospora anserina* . Mol Gen Genet 252: 539–551. 8914515 10.1007/BF02172400

[36] Choi GH, Larson TG, Nuss DL (1992) Molecular analysis of the laccase gene from the chestnut blight fungus and selective suppression of its expression in an isogenic hypovirulent strain. Mol Plant Microbe Interact 5: 119–128. 1535523 10.1094/mpmi-5-119

[37] Shapiro MB, Senepathy P (1987) RNA splice junctions of different classes of eukaryotes: sequence statistics and functional implications in gene expression. Nucleic Acids Res 15: 7155–7174. 3658675 10.1093/nar/15.17.7155 PMC306199

[38] Chung HJ, Kwon BR, Kim JM, Park SM, Park JK, et al (2008) A tannic acid-inducible and hypoviral-regulated laccase3 contributes to the virulence of the chestnut blight fungus *Cryphonectria parasitica* . Mol Plant Microbe In 21: 1582–1590. 10.1094/MPMI-21-12-1582 18986254

[39] Garavaglia S, Cambria MT, Miglio M, Ragusa S, Lacobazzi V, et al (2002) The structure of *Rigidoporus lignosus* laccase containing a full complement of copper ions, reveals an asymmetrical arrangement for the T3 copper pair. J Mol Biol 342: 1519–1531. 10.1016/j.jmb.2004.07.100 15364578

[40] Piontek K, Antorini M, Choinowsky T (2002) Crystal structure of a laccase from the fungus *Trametes versicolor* at 1.90-Å resolution containing a full complement of coppers. J Biol Chem 277: 37663–37669. 12163489 10.1074/jbc.M204571200

[41] Li H, Webb SP, Ivanic J, Jensen JH (2004) Determinants of the reactive reduction potentials of type-1 copper sites in proteins. J Am Chem Soc 126: 8010–8019. 15212551 10.1021/ja049345y

[42] Kallio JP, Gasparetti C, Andberg M, Boer H, Koivula A, et al (2011) Crystal structure of an ascomycete fungal laccase from *Thielavia arenaria* – common structural features of asco-laccases. FEBS J 278: 2283–2295. 21535408 10.1111/j.1742-4658.2011.08146.x

[43] Ge H, Gao Y, Hong Y, Zhang M, Xiao Y, et al (2010) Structure of native laccase B from *Trametes* sp. AH28–2. Acta Cryst F66: 254–258. 10.1107/S1744309110000084 PMC2833030 20208154

[44] Kallio JP, Auer S, Jänis J, Anderberg M, Kruus K, et al (2009) Structure-function studies of a *Melanocarpus albomyces* laccase suggest a pathway for oxidation or phenolic compounds. J Mol Biol 392: 895–909. 19563811 10.1016/j.jmb.2009.06.053

[45] German UA, Muller G, Hunziker PE, Lerch K (1988) Characterization of two allelic forms of *Neurospora crassa* laccase amino- and carboxyl-terminal processing of a precursor. J Biol Chem 263: 885–896. 2961749

[46] Bulter T, Alcalde M, Sieber V, Meinhold P, Schlachtbauer C, et al (2003) Functional expression of a fungal laccase in *Saccharomyces cerevisiae* by directed evolution. Appl Environ Microbiol 69: 987–995. 12571021 10.1128/AEM.69.2.987-995.2003 PMC143632

[47] Andberg M, Hakulinen N, Auer S, Saloheimo M, Koivula A, et al (2009) Essential role of the C-terminus in *Melanocarpus albomyces* laccase for enzyme production, catalytic properties and structure. FEBS J 276: 6285–6300. 19780817 10.1111/j.1742-4658.2009.07336.x

[48] Pardo I, Vicente AI, Mate DM, Alcalde M, Camareno S (2012) Development of chimeric laccases by directed evolution. Biotechnol Bioeng doi 10.1002/bit.24588. 10.1002/bit.24588 22729887

[49] Pezzella C, Autore F, Giardina P, Piscitelli A, Sannia G, et al (2009) The *Pleurotus ostreatus* laccase multi-gene family: isolation and heterologous expression of new family members. Curr Genet 55: 45–57. 19034452 10.1007/s00294-008-0221-y

[50] Hoegger PJ, Navarro-González M, Kilaru S, Hoffmann M, Westbrook ED, et al (2004) The laccase gene family in *Coprinopsis cinerea* (*Coprinus cinereus*). Curr Genet 45: 9–18. 14600788 10.1007/s00294-003-0452-x

[51] Nielsen H, Engelbrecht J, Brunak S, von Heijne G (1997) Identification of prokaryotic and eukaryotic signal peptides and prediction of their cleavage sites. Protein Eng 10: 1–6. 10.1093/protein/10.1.1 9051728

[52] Vite-Vallejo O, Palomares LA, Dantán-González E, Ayala-Castro HG, Martínez-Anaya C, et al (2009) The role of N-glycosylation on the enzymatic activity of a *Pycnoporus sanguineus* laccase. Enzyme Microb Technol 45: 233–239.

[53] Martin F, Laurent P, de Carvalho D, Voiblet C, Balestrini R, et al (1999) Cell wall proteins of the ectomycorrhizal basidiomycete *Pisolithus tinctorius*: identification, function and expression in symbiosis. Fungal Genet Biol 27: 161–174. 10441442 10.1006/fgbi.1999.1138

[54] Brandhorst T, Klein B (2000) Cell wall biogenesis of *Blastomyces dermatitidis*. Evidence for a novel mechanism of cell surface localization of a virulence-associated adhesin via extracellular release and reassociation with cell wall chitin. J Biol Chem 275: 7925–7934. 10713109 10.1074/jbc.275.11.7925

[55] Soragni E, Bolchi A, Balestrini R, Gambaretto C, Percudani R, et al (2001) A nutrient-regulated, dual localization phospholipase A_2_ in the symbiotic fungus *Tuber borchii* . The EMBO J 20: 5079–5090. 11566873 10.1093/emboj/20.18.5079 PMC125632

[56] Zhu X, Williamson PR (2004) Role of laccase in the biology and virulence of *Cryptococcus neoformans* . FEMS Yeast Res 5: 1–10. 15381117 10.1016/j.femsyr.2004.04.004

[57] Oda K, Kakizono D, Yamada O, Lefuji H, Akita O, et al (2006) Proteomic analysis of extracellular proteins from *Aspergillus oryzae* grown under submerged and solid-state culture conditions. Appl Environ Microbiol 72: 3448–3457. 16672490 10.1128/AEM.72.5.3448-3457.2006 PMC1472361

[58] Yogev O, Pines O (2011) Dual targeting of mitochondrial proteins: mechanism, regulation and function. Biochim Biophys Acta 1808: 1012–1020. 20637721 10.1016/j.bbamem.2010.07.004

[59] Ben-Menachem R, Tal M, Shadur T, Pines O (2011) A third of the yeast mitochondrial proteome is dual localized: a question of evolution. Proteomics 11: 4468–4476. 21910249 10.1002/pmic.201100199

[60] Regev-Rudzki N, Karniely S, Ben-Haim NN, Pines O (2005) Yeast aconitase in two locations and two metabolic pathways: seeing small amounts is believing. Mol Biol Cell 16: 4163–4171. 15975908 10.1091/mbc.E04-11-1028 PMC1196327

[61] Yogev O, Naamati A, Pines O (2011) Fumarase: a paradigm of dual targeting and dual localized functions. FEBS J 278: 4230–4242. 21929734 10.1111/j.1742-4658.2011.08359.x

[62] Vanhee C, Guillon S, Masquelier D, Degand H, Deleu M, et al (2011) A TSPO-related protein localizes to the early secretory pathway in *Arabidopsis*, but is targeted to mitochondria when expressed in yeast. J Exp Bot 62: 497–508. 20847098 10.1093/jxb/erq283 PMC3003801

[63] Byun-McKay SA, Geeta R (2007) Protein subcellular relocalization: a new perspective on the origin of novel genes. TRENDS Ecol Evol 22: 338–344. 17507112 10.1016/j.tree.2007.05.002

[64] Stohs SJ, Bagchi D (1995) Oxidative mechanisms in the toxicity of metal ions. Free Radic Biol Med 18: 321–336. 7744317 10.1016/0891-5849(94)00159-h

[65] Cobine PA, Ojeda LD, Rigby KM, Winge DR (2004) Yeast contain a non-proteinaceous pool of copper in the mitochondrial matrix. J Biol Chem 279: 14447–14455. 14729672 10.1074/jbc.M312693200

[66] Emanuelsson O, Brunak S, von Heijne G, Nielsen H (2007) Locating proteins in the cell using TargetP, SignalP and related tools. Nat Protoc 2: 953–971. 17446895 10.1038/nprot.2007.131

[67] Fitzpatrick DA (2012) Horizontal gene transfer in fungi. FEMS Microbiol Lett 329: 1–8. 22112233 10.1111/j.1574-6968.2011.02465.x

[68] Sprockett DD, Piontkivska H, Blackwood CB (2011) Evolutionary analysis of glycosyl hydrolase family 28 (GH28) suggests lineage-specific expansions in necrotrophic fungal pathogens. Gene 479: 29–36. 21354463 10.1016/j.gene.2011.02.009

